# Long-Term Follow-Up of Vestibular Function in Cochlear-Implanted Teenagers and Young Adults

**DOI:** 10.3390/audiolres15020042

**Published:** 2025-04-13

**Authors:** Niki Karpeta, Eva Karltorp, Luca Verrecchia, Maoli Duan

**Affiliations:** 1 Department of Clinical Science, Intervention and Technology, Karolinska Institute, 141 86 Stockholm, Sweden; eva.karltorp@ki.se (E.K.); luca.verrecchia@ki.se (L.V.); maoli.duan@ki.se (M.D.); 2 Department of Otolaryngology Head and Neck Surgery & Audiology and Neurotology, Karolinska University Hospital, 141 86 Stockholm, Sweden

**Keywords:** vestibular, children, TAYACI, adolescence, vHIT, VEMP, cochlear implantation, bilateral vestibulopathy, bilateral vestibular loss

## Abstract

Background: Vestibular function implements head position regulation and body spatial navigation. It matures during childhood and adolescence and integrates with the completion of an individual’s motor development. Nevertheless, vestibular impairment is associated with profound paediatric hearing loss and has a negative impact on the child’s motor proficiency. Cochlear implantation (CI) is the treatment of choice for severe hearing loss, where conservative treatment plans are not appropriate or fail. The Teenager and Young Adults Cochlear Implant (TAYACI) study investigates the long-term outcomes of early implantation with respect to the hearing, speech, psychological, and balance development among CI users. Methods: This study focuses on the vestibular function and the appropriate methods for vestibular assessment. The results of two established vestibular test methods are explored: the video head impulse test (vHIT) and cervical/ocular vestibular-evoked myogenic potentials (cVEMP, oVEMP) with air and bone conduction vibration stimulation. The results of vHIT, cVEMP, and oVEMP, per implanted ear and the relation to the aetiology of hearing loss are reported. An additional dynamic visual acuity (DVA) test was included to assess clinical oscillopsia. Results: Overall abnormal lateral canal testing was detected in 35/76 (46.1%) of the implanted ears. Bone-conducted cVEMP (BC cVEMP) was pathological in 33/76 (43.3%) and BC oVEMP in 42/76 (55.3%). Lateral canal impairment was associated with the background diagnosis of the hearing loss. Oscillopsia was related to bilateral canal impairment (sensitivity 73% specificity 100%). Conclusions: Lateral canal testing together with BC VEMPs were the most reproducible modules for vestibular testing The above tests were related to each other and complemented the overall vestibular assessment. DVA is a helpful tool to screen dynamic oscillopsia in patients with bilateral vestibular impairment.

## 1. Introduction

Approximately 200 children are born with some level of hearing loss every year in Sweden [1], and the prevalence of severe hearing loss is 0.2–0.3 per 1000 neonates in Stockholm [2,3]. A number of those will require cochlear implantation (CI). The aetiology of hearing loss can vary between different syndromes and unidentified causes, nevertheless requiring hearing rehabilitation. CI is a relatively minimal procedure and is considered safe, due to standardised surgical protocols. Indications for CI increase together with the improvement in hearing impairment diagnostics. This allows more children in need of rehabilitation to benefit from its use. Severe hearing loss shows a strong correlation to vestibular impairment in 40–60% of the cases [4] that is associated with delayed motor development and poor balance skills [5]. It is also argued that vestibular impairment in children is associated with weakened spatial ability and reduced cognitive performance in comparison to their peers with an intact vestibular system [6]. This state will persevere a long time after vestibular compensation or in the absence of appropriate intervention.

Vestibular impairment also affects the static and dynamic visual acuity (DVA) as the system fails to obtain stable images on the retina when the head is in motion, leading to blurred vision, oscillopsia, and reading difficulties during head movements. The DVA is not well investigated in children; however, an association between reduced DVA and underscored reading skills have been reported previously [7,8]. Moreover, research of vestibular function in the long term among adolescents with severe hearing loss and CI is sparse. Traditionally, studies on children who have received CI have focused on the associated speech delay and learning difficulties. Lately though, the interest in their overall performance in real environments has increased [9]. Both hearing and vestibular impairment are sensory deficits that affect the way a person interacts with its surroundings. In view of such deficits, and although the focus remains on sensory input restoration, better understanding of the long-term impact in morbidity and behavioural outcomes is of great importance. As a consequence, we designed The Teenage and Young Adults with CI study (TAYACI) [10], a multidisciplinary follow-up among CI users in adolescence. It consists of five ancillary studies that investigate the hearing, balance, speech, and psychosocial health outcome together with aetiological factors in teenagers with severe hearing loss who received CIs early in life.

In this study, we aimed to collect data on the vestibular function in the TAYACI group. All had been implanted as young children. We tested the canal function (anterior, lateral, and posterior) and the otolith function (sacculus and utriculus) of 38 teenagers, and we examined all five parts of the vestibular system in each ear separately. The DVA was used as an additional functional measurement of vestibular impairment. This report focuses on the following:

1. Describing the vestibular function in the long-term;

2. Exploring which clinical tests could possibly be used as vestibular screening;

3. Identifying patients with reduced DVA and bilateral vestibular impairment with a simple, accessible test.

## 2. Materials and Methods

Study design

Cross-sectional study

Materials

Teenagers with CI aged between 11 and 20 years in our hospital’s CI registry were recruited through invitation. They had been diagnosed with severe hearing loss/deafness as newborns and had been implanted bilaterally early in life (mean age 15.4 months). The patients were tested 10–19 years after their last surgical procedure. Thirty-eight patients consented to participate in vestibular testing, and they were age-matched with 20 peers with normal hearing. All CI participants but one had bilateral cochlear implants. The participants were tested at our tertiary referral audiology and neurotology department at Karolinska University Hospital in Stockholm between March 2022 and June 2023, and the study was approved by the National Ethical Committee (n 2021-04345). Written informed consent was obtained by all study participants. Parental consent was obtained when the participant was a minor.

Methods

Two established vestibular test methods were used: the video head impulse test (vHIT) and cervical/ocular vestibular-evoked myogenic potentials (cVEMP, oVEMP) with air-conduced (AC) and bone-conducted (BC) stimulation. These methods enabled us to test all five parts of the vestibular system. We tested the saccular, utricular, and all three canals’ function in each ear, one side at a time. An additional visual acuity test (DVA) was included after the vHIT and VEMP examinations. Before testing, patients removed the external CI units.

vHIT protocol

The routine for vHIT consisted of the measurement of the vestibulo-ocular reflex (VOR) gain, in response to head thrusts on the plane of canal stimulation, with an ICS^®^ Impulse with Otosuite vestibular software v.4.31 (Natus Medical Denmark ApS, Taastrup, Denmark).

At least 15 approved repetitions were required in yaw plane and 10 in the LARP and RALP planes, respectively. A gain value between 0.8 and 1.0 in the lateral plane and between 0.7 and 1.0 in the vertical planes with no saccades was considered normal [11,12]. Gain with borderline values (between 0.7 and 0.8) were considered normal or pathological in the absence or presence of refixation saccades with reference to the values of the control group.

VEMP protocol

For VEMP testing, we used an eclipse-evoked potential system with VEMP module (Interacoustics A/S, Middlefart, Denmark with EPx5 software v 4.6.1). VEMP recordings were reproduced with AC and BC stimulation. AC stimulation was conducted with plug-in headphones while BC was conducted with placement of a Radioear B81 bone transducer on the mastoid. One ear was tested at a time. The stimulus was a tone burst at 500 Hz (2-1-2 ms) at 132 peSPL for air-conducted (AC VEMP) stimulus and 135 peSPL for bone-conducted (BC VEMP) cervical testing. A total of two repetitions of 100 sweeps (test–retest) were performed on each side. The device permitted an EMG-controlled stimulation delivery protocol: stimuli were presented only when the EMG levels recorded on cervical electrodes during active head elevation in supine position were between 50 and 150 microvolts. This enabled us to standardise the muscle activation in the tested subjects and avoid the known bias of cVEMP amplitude variation related to the level of sternocleidomastoid muscle activation. The cVEMP amplitude was furthermore expressed as scaled amplitude on the EMG pre-stimulus, according to the device protocol. In ocular VEMPs (oVEMP), the active electrodes were placed on the outer part of each lower eyelid and a contralateral reflex was recorded from the inferior oblique muscle. The patients were sitting and looking upwards with their gaze at a 30 degree angle, staring at a point on the ceiling, and 100 sweeps were collected twice on each side by default (test–retest). For VEMP testing, we assessed response wave reproducibility (WR), amplitudes, and latencies in all tested ears. Responses in the CI group with WR within the 5–95% WR percentile range were considered normal and in reference to the NH group. Amplitudes and latencies of the identified waves were calculated. 

DVA protocol

Lastly, the patients were tested with a DVA test [13]. We used a standard optometric chart, and the patients sat two meters away wearing their prescription glasses when required. They were instructed to read the letters on the chart lines from top to bottom until they could no longer identify all the letters on a given line. The chart line above that was considered as baseline for the specific patient. Then the examiner rotated the patient’s head with a frequency of 2 Hz, with two head rotations per second, at a 60 degree angle in the yaw plane. The test was abnormal if the visual acuity decreased by at least three rows during the examination.

All CI recipients were tested by two specialists who were blinded regarding the patients’ diagnosis of hearing loss. Both CI and NH teenagers were tested with the same equipment as per the protocol.

Data Analysis and Statistics

The vHIT and VEMP results refer to ‘’number of ears’’ and not ‘’number of patients’’, as vestibular function can differ between the left and right ear. The DVA test results refer to number of patients, with testing VOR on the lateral canal plane in a frequency range in which both sides contribute to gaze stabilisation.

Our data were not normally distributed and, for the purpose of the analysis of the canal and otolithic function, we report median values and interquartile range (IQR) for these variables. We used a nonparametric test (Mann–Whitney) to analyse and compare the vestibular function of CI recipients vs. normal hearing controls (NH). We analysed vHIT gain values for all canals. For VEMP results, we studied wave reproducibility (WR), wave amplitudes (in microvolts), and latencies. WR indicates test reliability in VEMP measurements. It is obtained by the cross-correlation between two banks of collected responses calculated by the device. A normative value is considered the lower limit of the confidence interval calculated in the control group. Pathological vs. normal responses were related to normative data with 95% confidence interval. Scaled amplitudes were used only for cVEMP results. A power analysis based on the distribution of vHIT results (lateral canals) in the case group and control group showed that the final cohort of 38 CI and 20 controls was adequate for statistical inference. A one-way ANOVA was used to study the VOR gain distribution in relation to the diagnosis of deafness. Post hoc tests defined the difference in mean VOR gains between the singular diagnostic groups (Supplementary Materials).

## 3. Results

The age distribution did not differ significantly between the invited TAYACI (n = 108) and the accepting-to-participate group (n = 53). Thirty-eight of fifty-three came for vestibular testing, and age distribution differed significantly between these groups: individuals in the accepting group were on average 1.18 years older (*p* = 0.04). This discrepancy was considered not clinically relevant given the large age span of the tested cohort (11–20) Gender distribution was not different between groups (female:male = 1.12 in accepting vs. 1.11 in the vestibular group).

Our group presented with a heterogeneity of background diagnoses associated with hearing loss, shown in Table 1. In most cases though (9 patients), no apparent cause of profound hearing loss could be identified.

vHIT testing results

**Figure 1:**
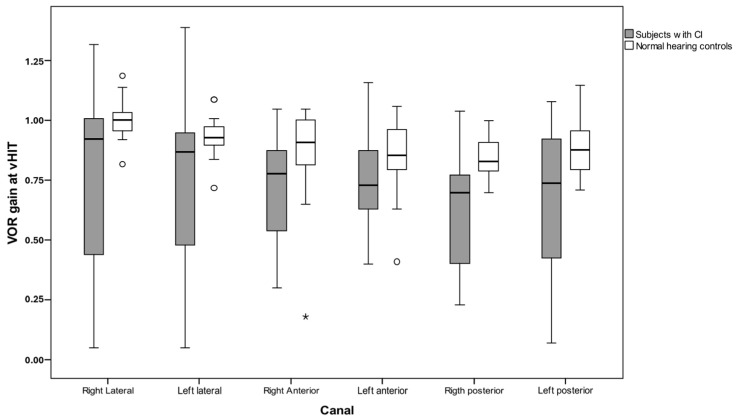
Six canal gain distribution per side in CI and NH. Circles and ^*^ represent outliers.

The VOR gain distribution of each canal in CI patients vs. NH controls is shown in Figure 1. Gain values in the implanted group were scattered in all tested canals compared to normal peers.

**Figure 2:**
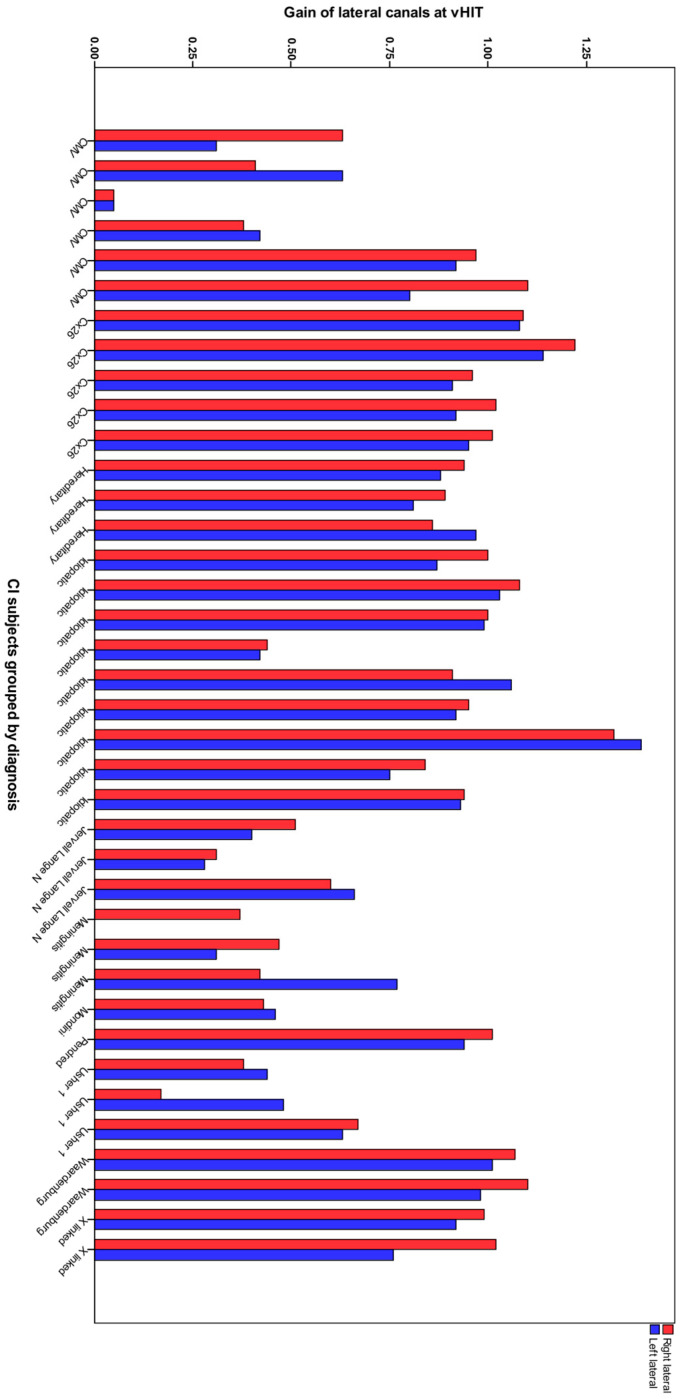
Lateral canal gain per ear and diagnosis in CI recipients.

Gain distribution between the two groups showed a statistically significant difference in all canals and always in favour of the NH group. Only one of the controls presented with pathological canal gains, and that was unilateral. Fourteen of the thirty-eight CI recipients had pathological lateral canal gain bilaterally. No CI recipient was found with a unilateral pathological lateral gain. Only one patient had a unilateral right-sided implant. This recipient had a meningitis diagnosis and a bilateral pathological lateral canal gain. Figure 2 shows the relation of lateral canal gain and diagnosis in tested CI ears. Median gain distribution of all six canals and IQR in CI and NH was also calculated and, these data are shown in Table 2. Mean gain was statistically significantly different between different diagnoses; Welch’s F (10, 14.459) = 52.427, *p* < 0.001. Games-Howell post hoc analysis conducted between the different diagnostic groups showed stratification in the two groups according to diagnosis. Lateral canal gains relative to Usher, CMV, Mondini, Meningitis, and Jervell Lange were significantly lower compared to Cx26, Waardenburg, X-Linked, Pendred, hereditary, and idiopathic group. The two groups could be separated by a cut-off value of 0.6 in lateral canal gain.

**Figure 3:**
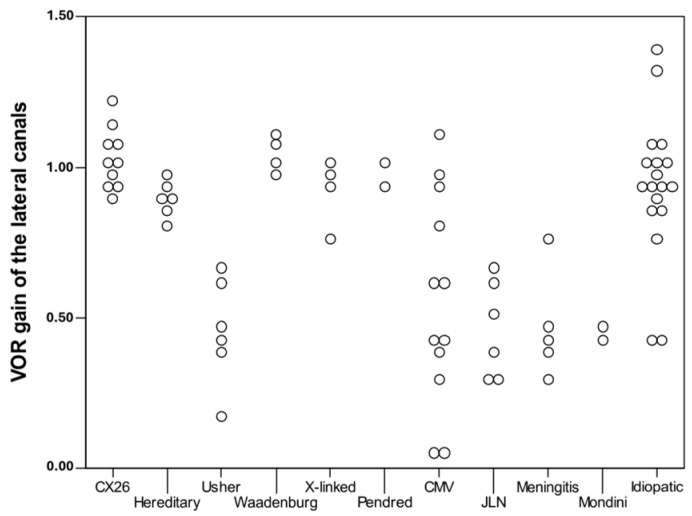
Relation between diagnosis and gain of lateral canals (per ear).

To determine whether the canal gain was statistically related to the cause of the hearing loss, we explored the distribution of lateral gain values of different diagnoses. CI ears with a background diagnosis of meningitis, Usher, and Jervell Lange-Nielsen syndrome (JLN) showed worse gain values, whereas patients with congenital cytomegalovirus (cCMV) infection presented variability from normal to complete canal failure (Figure 3).

DVA testing

Outcomes were analysed in relation to bilateral lateral canal VOR with vHIT. The test demonstrated 100% specificity (20/20) and 73.3% sensitivity (11/15) (χ^2^ = 24.6, *p* < 0.001).

VEMP testing results

Descriptive analysis, including WR, amplitudes, and latencies with their median values and interquartile range (IQR) of the tested modules, is shown in Table 3. In one CI patient (two ears), no VEMP measures could be recorded due to technical issues.

Wave reproducibility (WR) was a decisive factor in the result interpretation. Normal controls showed a high WR (97.3–100%) in all modules except AC oVEMP (65.9–83.3%), and this module was excluded from further analysis. Mann–Whitney test in CI recipients showed a significantly lower WR in all VEMP modules in comparison to normal hearing peers (*p* < 0.001). The VEMP waves of the CI group within the normative WR range of 5–95 percentile were present in 37 ears (49.3%) of AC cVEMP, in 42 ears (56%) of BC cVEMP, and 46 ears (61.3%) of Bc oVEMP. The proportion of cVEMP responses with WR within the normative range was not significantly different between AC and BC cVEMP testing (Pearson chi-square, *p* > 0.05).

In those reproducible waves, we analysed amplitudes and latencies and compared them to the NH.

Amplitudes. The scaled amplitudes in the AC cVEMP testing were significantly lower in the CI group compared to NH (*p* < 0.001), whereas there was not a statistical difference between the two groups in the BC cVEMP test (*p* > 0.05). Additionally, the absolute amplitudes of the reproducible responses in the BC oVEMP tests showed no difference in distribution between the two groups (*p* > 0.05).

Latencies. In AC cVEMP, the distribution of P1 peak latencies showed no difference between the two groups (*p* > 0.05), whereas the distribution of N1 peak latency showed a *p* = 0.034, with CI patients showing earlier peak responses. There was no significant difference in distribution of P1 and N1 latencies in the BCcVEMP reproducible responses. BC oVEMP:

N1 values showed delayed peak responses in the CI group (*p* = 0.04), but that was not true for the P1 peak values (*p* > 0.05).

Relation between vHIT, VEMP, and DVA.

Overall abnormal lateral canal testing was detected in 35/76 (46.1%); BC cVEMP was pathological in 33/76 (43.3%) and Bc oVEMP in 42/76 (55.3%) of the tested ears.

An χ^2^ analysis of the pathological vHIT and BC VEMP responses in the tested ears showed consistent results in the majority of cases. When vHIT showed pathological gain, BC VEMP was also pathological (*p* < 0.001). In particular, BC cVEMP was pathological in 23 (65.7%) of 35 ears with pathological vHIT. Respectively, Bc oVEMP was pathological in 27/35 (77.1%). The same applied for the χ^2^ analysis of vHIT and DVA testing. DVA was never pathological when vHIT was normal.

## 4. Discussion

This paper describes the long-term vestibular function in cochlear-implanted ears. We tested all five parts (three canals, sacculus, and utriculus) in each ear, and we found that CI recipients showed compromised vestibular responses in at least one of the tested modules.

VEMP responses were inferior in CI recipients compared to their NH peers. This was detected not only in terms of amplitude and latency responses but also in terms of WR, indicating that we should expect weaker and fewer replies in CI ears. To our surprise, WR in AC oVEMP testing was low and widespread in the healthy controls as well (median 83.5 IQR 32.75). We assumed that this group would have normal otolithic responses and considered these results as a main technical issue. We thus concluded that AC oVEMP was not a reliable module for otolithic function testing in this age group and was excluded from further analyses. We did not find a statistically significant difference in WR between AC cVEMP and BC cVEMP and CI and NH groups. Though, we found lower AC cVEMP amplitudes and earlier N1 responses in the CI compared to the NH group. This result is consistent with decreased AC cVEMP amplitudes reported in CI recipients in a previous study in 2009 [14]. However, BC VEMP responses in our study did not support this. In fact, normal BC VEMP responses in patients with pathological AC VEMP suggested that the latter may propose false positive vestibular impairment. A possible explanation would be that AC cVEMP responses may be affected by the presence of the CI electrode array acting as a foreign body in the inner ear. The predisposition of AC cVEMP compared to BC cVEMP post-CI has been discussed in other clinical studies in adult populations [15]. It seems that BC cVEMP is more stable as testing method and thus favoured in the clinical praxis of the vestibular assessment in younger patient groups [16], and our results are consistent with this.

In our study, vHIT results showed pathological lateral canal gains in 36% (28/78) of the tested ears and a decreased gain in one or more of the vertical canals in 74%, (58/78). Similar results have been reported in a study in 2005 [17]. The estimated risk of an absolute or a partial vestibular impairment in children and adults after cochlear implantation is reported between 0 and 77%, with most studies reporting caloric results post-op [9,18,19,20,21,22]. Gurkov, in an article in 2009 [23], argues that cochlear implantation is a risk for the function of the lateral canals, based on the worsening of caloric responses postoperatively in an adult population. However, Simon [24] recently reported that “CI electrode-array interferes with caloric examination and overestimates post-implantation vestibular deficits” in a study of 162 children. He reported only 27 ears with vestibular deterioration after surgery, suggesting that calorics tend to overestimate vestibular trauma. This supports our initial hypothesis about the electrode presence overrating vestibular impairment as mentioned before, with AC stimulation in VEMP measurements. In fact, if AC cVEMP and oVEMP modules were not taken into account, we could find nine CI recipients (18 ears) with both vHIT and BC VEMP approved responses. Thus, we propose vHIT for canal function and BC VEMP for saccular and utricular testing.

Additionally, we observed that pathological vHIT and VEMP results were related to each other even though they are testing methods for different parts of the vestibular system. Although it is always preferable to test patients with more than one method, we consider this knowledge significant in cases where this is impossible due to clinical impediments.

The present study is retrospective and singlecentred and considers a small sample size. The majority were operated on with a cochleostomy (53/75 ears), which tends to be more traumatic for the vestibulum compared to the round window approach [25,26]. A study limitation is that we lacked information on the vestibular function preoperatively, as this kind of testing was not included in the patients’ preoperative assessment in the early 2000s. However, our aim was to present the actual vestibular function in adolescence where integration and completion of motor development occurs. A future multicentred study incorporating preoperative vestibular assessment and longitudinal follow-up of CI recipients would contribute to a better understanding of the progress of vestibular function over time in these patients.

Measurable vestibular impairment following CI has been described in several other papers [20,27,28,29]. However, they do not show homogeneity in terms of population or time of testing. It has been argued that some patients show only transient vestibular impairment postoperatively [30]. In a recent scoping review including 14 articles published in 2022 [28], the vestibular function in CI children was explored and found to be preserved or even restored partially after the operation. Our results are comparable, as most of the tested ears showed function within normal ranges in one or more tested modules. Only 18 ears showed both normal gain in all three canals on vHIT and normal BC VEMP responses.

vHIT and VEMPs are clinical tools for assessing vestibular impairment. It is known that bilateral vestibular loss is associated with dynamic oscillopsia, the oscillating sensation of one’s surroundings while moving. Therefore, we added DVA testing in our protocol. We observed that this was an easy and quick method of screening CI recipients with clinical oscillopsia related to severe bilateral VOR impairment. This test can document clinical impairment, which can be different to a measured vestibular loss [31] by vHIT and VEMP, as it involves the subject’s compensation ability. We found that, when DVA was pathological, the gain of the lateral canals was always pathological bilaterally (gain < 0.6). Interestingly, some cCMV patients with bilateral pathological gain results demonstrated a normal DVA, a result that implies that some individuals present with better compensatory behaviours. We consider this as an interesting point for future investigation, as understanding the mechanisms behind compensation is crucial for clinical management and rehabilitation strategies.

The vestibular system is already functional by birth, but its central connections can still develop during adolescence. Taking this into consideration, we saw the need for a control group, age- and sex-matched to CI recipients. We recruited 20 healthy adolescents based on a power analysis of vHIT results. Curthoys et al., in 2015 [32] in an article on age-dependent VOR gain values, argues that gain is largely unaffected by age, although vertical canal gain showed greater variability. Another study in 2021 [11], which included 100 teenagers, showed similar results with mean values within normal range (0.8–1.0). The reference values in our control group were consistent with the above studies.

Recent studies in children have estimated a 2% risk of vestibular impairment following CI [22,33,34]. Although this risk is not negligible, it is much lower than the frequency of vestibular impairment linked to the underlying aetiology of hearing loss that leads to CI [4]. Idiopathic hearing loss was the diagnosis of our largest subgroup (nine patients), followed by cCMV (six patients), and CX26 (five patients), and our findings regarding vestibular impairment were consistent with those reported in the literature, supporting a strong relation to the aetiology of the severe hearing loss/deafness [35,36,37]. We could not retrieve information of the different subtypes of Waardenburg syndrome diagnosis in the patients’ notes. The hereditary group consisted of CI recipients where inheritance was suspected but could not be confirmed with genetic analysis. Syndromes like Usher I or JLN always demonstrated pathological gains on vHIT, whereas CX26 and idiopathic hearing loss was not associated with severe vestibular impairment, and patients with such diagnoses presented with normal or close-to-normal gains on vHIT. Surprisingly, lateral canal gains in cCMV patients ranged between zero to normal values, despite our initial predictions that these patients would score badly. Vestibular assessment is always recommended in cCMV patients though, because vestibular defect can exist and progress without any hearing loss [4,38,39]. We believe this is important information to provide to our present and future patients.

In summary, this study is part of a larger multidisciplinary research programme designed to understand the long-term effects of cochlear implantation in adolescents and young adults (TAYACI). The target group included patients who were operated with CI early in life and were followed up regarding cognition, linguistic abilities, psychological development, hearing, and balance. This paper, though, focuses only on the continuance and preservation of the vestibular function in the above group. Data were gathered within 15 months and the post-op time span, at the time of vestibular testing, which was between 10 and 19 years. Abnormal lateral canal testing was detected in 46.1%, whereas BC cVEMP was pathological in 43.3% and Bc oVEMP in 55.3 of the tested ears. Pathological results were associated with syndromic diagnoses, something supported by the international literature [9]. Similarly, in diagnoses (CX26, idiopathic) where the vestibular system tended to be spared, we could demonstrate good function, supporting our hypothesis that CI is a quite safe procedure, and vestibular function can be preserved in the long term. Our results support a strong relation between BC VEMP, vHIT, and DVA, and we propose that a protocol based on these tests may represent the best way to define the integrity of vestibular pathways in CI recipients.

## 5. Conclusions

This research paper has explored the results of vHIT and VEMP in adolescents operated on with CI at a young age, shedding light to their vestibular responses. The above tests were related to each other and complemented the overall vestibular assessment. Finally, the addition of a DVA test contributed to a preliminary estimation of a patient’s oscillopsia related to severe bilateral canal impairment.

## Figures and Tables

**Table 1:** Underlying diagnosis in CI patients.

Diagnoses	n. of Patients
Idiopathic (unknown)	9
Congenital Cytomegalovirus (cCMV)	6
CX26 (connexin 26)	5
Usher I	3
Jervell and Lange-Nielsen	3
Meningitis	3
Hereditary	3
Waardenburg syndrome	2
X-linked deafness	2
Pendred syndrome	1
Mondini Dysplasia	1

**Table 2:** Canal gain distribution between CI patients and NH controls (Mann–Whitney test).

Canals	CI Median Gain (IQR)	NH Median Gain (IQR)	*p*
RA	0.78 (0.35)	0.91 (0.20)	0.002
RL	0.92 (0.58)	1.00 (0.08)	0.015
RP	0.70 (0.40)	0.83 (0.14)	0.000
LA	0.73 (0.28)	0.85 (0.18)	0.019
LL	0.87 (0.49)	0.93 (0.08)	0.031
LP	0.74 (0.51)	0.88 (0.17)	0.005

RA: right anterior; RL: right lateral; RP: right posterior; LA: left anterior; LL: left lateral; LP: left posterior; CI: cochlear implant; NH: normal hearing.

**Table 3:** Comparison of VEMP modules (Mann–Whitney test) between cochlear implanted (CI) and normal hearing controls (NH).

	CI	NH		CI	NH		CI	NH		CI	NH	
	WR	WR	*p*	AMPL	AMPL	*p*	P1	P1	*p*	N1	N1	*p*
	Median (IQR)	Median (IQR)		Median (IQR)	Median (IQR)		Median (IQR)	Median (IQR)		Median (IQR)	Median (IQR)	
AC cVEMP	53.5 (58.5)	97.0 (9.5)	<0.001	0.4 (0.7)	1.2 (1.32)	<0.001	14.7 (2.6)	14.7 (1.2)	0.94	23.8 (3.1)	25.3 (4.0)	0.03
BC cVEMP	79.5 (73.2)	97.0 (8.0)	<0.001	1.2 (1.5)	1.5 (1.34)	0.17	14.7 (2.3)	15.3 (2.1)	0.34	24.7 (3.3)	25.3 (3.0)	0.42
AC oVEMP	43.0 (43.5)	83.5 (32.8)	<0.001	0.0 (5.2)	6.4 (6.7)	n/a	16.5 (4.4)	16.3 (1.0)	n/a	11.0 (1.5)	11.7 (1.3)	n/a
BC oVEMP	70.0 (50.0)	97.0 (4.0)	<0.001	9.8 (14.3)	18.4 (16.3)	0.06	16.3 (1.0)	16.0 (1.7)	0.06	11.5 (1.1)	11.3 (0.8)	0.04

n/a: not available.

## Data Availability

The data presented in this study are available on request from the corresponding author due to personal data protection reasons (personal patient data).

## Supplementary Materials

The following supporting information can be downloaded at: https://www.mdpi.com/article/10.3390/audiolres15020042/s1.
