# Supplementary material for: Long-Term Follow-Up of Vestibular Function in Cochlear-Implanted Teenagers and Young Adults
Source: Audiol Res. 2025 Apr 13;15(2):42. doi: 10.3390/audiolres15020042 (PMC12024341; doi:10.3390/audiolres15020042)
Supplement: Supplementary file 1 [file audiolres-15-00042-s001.zip › audiolres-3536806-supplementary.pdf]

Supplement

The relation between the VOR gain of lateral canal and diagnosis.

A one-way Welch ANOVA was conducted to determine if the VOR gain of the lateral canal was different for different diagnoses. Mean VOR gain was statistically significantly different between different diagnoses, Welch's $F(10, 14.459) = 52,427$, $p < 0.001$. Games-Howell post hoc analysis was conducted between the 11 different diagnostic classes:

Descriptives

Gain_lat

	N	Mean	Std. Deviation	Std. Error	95% Confidence Interval for Mean		Minimum	Maximum
					Lower Bound	Upper Bound		
Cx26	10	1,0300	,10143	,03208	,9574	1,1026	,91	1,22
Usher	6	,4617	,18126	,07400	,2714	,6519	,17	,67
CMV	12	,5558	,34558	,09976	,3363	,7754	,05	1,10
Waardenburg	4	1,0400	,05477	,02739	,9528	1,1272	,98	1,10
Hereditary	6	,8917	,05707	,02330	,8318	,9516	,81	,97
Idiopathic	18	,9356	,23902	,05634	,8167	1,0544	,42	1,39
Mondini	2	,4450	,02121	,01500	,2544	,6356	,43	,46
X-linked	4	,9225	,11615	,05808	,7377	1,1073	,76	1,02
Menigitis	5	,4680	,17894	,08002	,2458	,6902	,31	,77
Pendred	2	,9750	,04950	,03500	,5303	1,4197	,94	1,01
Jervell Lange	6	,4600	,15531	,06340	,2970	,6230	,28	,66
Total	75	,7696	,30648	,03539	,6991	,8401	,05	1,39

Table XY Descriptive statistics: mean gain of lateral canals for each diagnostic class.

	Cx26	Usher	CMV	Waardenburg	Hereditary	Idiopathic	Mondini	X-linked	Menigitis	Pendred	Jervell Lange
Cx26		-,56833*	-,47417*	,01000	-,13833	-,09444	-,58500*	-,10750	-,56200*	-,05500	-,57000*
Usher	,56833*		,09417	,57833*	,43000*	,47389*	-,01667	,46083*	,00633	,51333*	-,00167
CMV	,47417*	-,09417		,48417*	,33583	,37972	-,11083	,36667	-,08783	,41917*	-,09583
Waardenburg	-,01000	-,57833*	-,48417*		-,14833	-,10444	-,59500*	-,11750	-,57200*	-,06500	-,58000*
Hereditary	,13833	-,43000*	-,33583	,14833		,04389	-,44667*	,03083	-,42367	,08333	-,43167*
Idiopathic	,09444	-,47389*	-,37972	,10444	-,04389		-,49056*	-,01306	-,46756*	,03944	-,47556*
Mondini	,58500*	,01667	,11083	,59500*	,44667*	,49056*		,47750*	,02300	,53000	,01500
X-linked	,10750	-,46083*	-,36667	,11750	-,03083	,01306	-,47750*		-,45450*	,05250	-,46250*
Menigitis	,56200*	-,00633	,08783	,57200*	,42367	,46756*	-,02300	,45450*		,50700*	-,00800
Pendred	,05500	-,51333*	-,41917*	,06500	-,08333	-,03944	-,53000	-,05250	-,50700*		-,51500*
Jervell Lange	,57000*	,00167	,09583	,58000*	,43167*	,47556*	-,01500	,46250*	,00800	,51500*	

Table XX. difference in mean VOR gain between the different diagnostic classes, post hoc tests (Games-Howell). Starred mean VOR differences are stastistical significant

VOR gain values stratify significantly in two groups according to the diagnosis (table XY). The post-hoc analysis shows that mean VOR gains relative to Usher, CMV, Mondini, Meningitis and Jervell Lange are significantly lower than those in Cx 26, Waadernburg, hereditary and idiopathic forms, X-Linked and

Pendred groups. The differences are statistical significant and the two VOR gain groups can be separated by a cut off value around 0,6 in VOR gain. (Table XX)
